# Supplementary material for: Metagenomic discovery and co-infection of diverse wobbly possum disease viruses and a novel hepacivirus in Australian brushtail possums
Source: One Health Outlook. 2019 Dec 12;1:5. doi: 10.1186/s42522-019-0006-x (PMC7990097; doi:10.1186/s42522-019-0006-x)
Supplement: Supplementary file 1 — Additional file 1: Table S1. PCR primers and qPCR primers for WPDV used in this study. [file 42522_2019_6_MOESM1_ESM.pdf]

Table S1. PCR primers and qPCR primers for WPDV used in this study.

Targeted region	PCR assay	Primer sequence (5'-3')	Purpose
WPDV replicase polyprotein	WPDV_case_PCR1_F	TACACAACAGCAGTTAGTGG	Initial WPDV case checking (available for AU1)
	WPDV_case_PCR1_R	GTAAACTTCATTCACAAGAAGCC	
WPDV replicase polyprotein	WPDV_case_PCR2_F	GACACCGGTTGAGTCAAC	Initial WPDV case checking
	WPDV_case_PCR2_R	ATTCCAAGGATCTCGCAAAG	
WPDV replicase polyprotein	WPDV_case_PCR3_F	TAATCCGGTGAATGCCATTAC	Initial WPDV case checking
	WPDV_case_PCR3_R	CTATTACTACGACTGTCCGTG	
PCR1 (replicase polyprotein1ab) 471-4471	WPDV_AU1_PCR1_F	CCTTATTGTGACTTTGACATGGATG	Genome recovery for WPDV AU1
	WPDV_AU1_PCR1_R	GAGAGCCAGTACCGTATGACC	
PCR 2 (replicase polyprotein1ab) 2916-7142	WPDV_AU1_PCR2_F	GTGTCCTGTGCTTCAAGTGC	Genome recovery for WPDV AU1
	WPDV_AU1_PCR2_R	ATCAACATCAGGAAGTGCTTGC	
PCR 3 (replicase polyprotein 1ab) 6614-9811	WPDV_AU1_PCR3_F	CTTGCGTACTGTCGGGTATC	Genome recovery for WPDV AU1
	WPDV_AU1_PCR3_R	GACAAGGACTGGTTACATGGTG	
PCR 4 (replicase polyprotein 1ab, glycoprotein 2,3 and 4) 8622-11643	WPDV_AU1_PCR4_F	GCATCCTCATCATACACTTGCG	Genome recovery for WPDV AU1
	WPDV_AU1_PCR4_R	TCTGATGTCCAATATCCATAGTAAGG	
gap filling PCR 1 (replicase polyprotein 1ab) 1455-3074	WPDV_AU1_G1_F	CGGATGTGCTAGCTTTGTG	Gap filling of WPDV AU1
	WPDV_AU1_G1_R	GTGTGACAACCTGCAATGC	
gap filling PCR 2 4300-6162	WPDV_AU1_G2_F	AAGGCTGCTTACGATTCAG	Gap filling of WPDV AU1
	WPDV_AU1_G2_R	GAAGCTTCTCAACATCCTTG	
gap filling PCR 3	WPDV_AU1_G3_F	GTGACCTCCTTATCAAATTGTG	Gap filling of WPDV AU1

7500-8776	WPDV_AU1_G3_R	GCAGTACTGGTAGAACTTGC	
gap filling PCR 4 9653-10679	WPDV_AU1_G4_F	CGTTGTTACACCTCGCTC	Gap filling of WPDV AU1
	WPDV_AU1_G4_R	CAATGTATCTGAAATGAGTGCG	
PCR1 (replicase polyprotein1ab) 476-4459	WPDV_AU2_PCR1_F	CTCTGATTTTGACATTGATGAGA CAG	Genome recovery for WPDV AU2
	WPDV_AU2_PCR1_R	CATGAGTCCATCCTGAAGCAG	
PCR 2 (replicase polyprotein1ab) 2847-7142	WPDV_AU2_PCR2_F	TAGCAGTCTTCGCATCATTACC	Genome recovery for WPDV AU2
	WPDV_AU2_PCR2_R	GTCAATGTTAGGTAGAGCTTGC	
PCR 3 (replicase polyprotein 1ab) 6610-9809	WPDV_AU2_PCR3_F	GTGGTCTTATGTGTGTTAACTAC C	Genome recovery for WPDV AU2
	WPDV_AU2_PCR3_R	CAAGGGCTAGTGACGTGATG	
PCR 4 (replicase polyprotein 1ab, glycoprotein 2,3 and 4) 8812-11614	WPDV_AU2_PCR4_F	CCAGAATCCACGTCACGCTC	Genome recovery for WPDV AU2
	WPDV_AU2_PCR4_R	GCATATTCCAGGTCTATAAGTGC C	
gap filling PCR 1 (replicase polyprotein 1ab) 1457-2981	WPDV_AU2_G1_F	CTGTGCTAGCTTTGTCAGG	Gap filling of WPDV AU2
	WPDV_AU2_G1_R	CACGACGATGTTCTTCACC	
gap filling PCR 2 4300-6162	WPDV_AU2_G2_F	CTTAAGGCTGCTTATGATTCAG	Gap filling of WPDV AU2
	WPDV_AU2_G2_R	GATTGCCTGTCTCAGTTTCTC	
gap filling PCR 3 7500-8776	WPDV_AU2_G3_F	CAACCCTCTCTAATTGTGTATAC	Gap filling of WPDV AU2
	WPDV_AU2_G3_R	ACGTCCCATTGGTTGCAG	
gap filling PCR 4 9653-10679	WPDV_AU2_G4_F	GTTGTCATCACGTCACTAGC	Gap filling of WPDV AU2
	WPDV_AU2 _G4_R	TCAGCATTGCCGATCATC	
3'UTR extension PCR	WPDV_AU1_end_F	GCCATCATCAAAGCAATAATTGA CCTGGA	Recovery of 3'UTR for WPDV AU1 and AU2
	WPDV_AU2_end_F	GTTCAGTGGAGGTATGGCAGCT G	
	WPDV_end_UTR_R	CACTATGCACGGACAGTCGTAG	

WPDV conserved RdRp catalytic domain	WPDV_qPCR_Pol_F	CGCAYTGAGATTAAAYTATCGACC	WPDV qPCR
	WPDV_qPCR_Pol_R	CAATCAGTRTGYAGTACCACG	
	WPDV_qPCR_Pol_P	/56-FAM/TGGGTCCCA/ZEN/GCACTHATTTATGGTGA/3IABkFQ/	
Hepacivirus Polyprotein 8643-6579	Possum_hepaci_PCR1_F	TCCAATTACCCAGCTTTGTG	Initial hepacivirus confirmation
	Possum_hepaci_PCR1_R	GGTCTTCCACTCAGTCAGTAG	
Hepacivirus Polyprotein 8663-6618	Possum_hepaci_PCR2_F	GGTCTCACGTCTACTGGC	Initial hepacivirus confirmation
	Possum_hepaci_PCR2_R	TACACCACGTACTTTCCCCA	
Hepacivirus-polyprotein	Possum_hepaci_PCR3_F	GAGGCAATTGAATTTGACTGG	Gap filling
	Possum_hepaci_PCR3_R	TGGAGTGTACATGTCTATTGAC	
Hepacivirus-polyprotein	Possum_hepaci_PCR4_F	ACATCTGTGTTCATGATGGTG	Gap filling
	Possum_hepaci_PCR4_R	ACAGATCACACAGGTAAATGG	
