# Supplementary material for: Metagenomic discovery and co-infection of diverse wobbly possum disease viruses and a novel hepacivirus in Australian brushtail possums
Source: One Health Outlook. 2019 Dec 12;1:5. doi: 10.1186/s42522-019-0006-x (PMC7990097; doi:10.1186/s42522-019-0006-x)
Supplement: Supplementary file 2 — Additional file 2: Table S2. Presentation and pathology of wobbly possum disease in brushtail possums in Australia. This table chronologically summarises the clinical signs and histological findings in brushtail possums considered to fit the syndrome description for wobbly possum disease. Most possums originate from New South Wales on mainland Australia, except for those denoted with a T that originate from Tasmania. [file 42522_2019_6_MOESM2_ESM.pdf]

Table S2. Presentation and pathology of Wobbly Possum Disease in brushtail possums in Australia. This table chronologically summarises the clinical signs and histological findings in brushtail possums considered to fit the syndrome description for Wobbly Possum Disease. Most possums originate from New South Wales on mainland Australia, except for those denoted with a "T" that originated in Tasmania.

				Degree of Lymphoplasmacytic Inflammation							
**Case ID**	**Date**	**Signalment**	**Presentation**	**Meninges**	**Cerebrum**	**Brain stem**	**Cerebellum**	**Optic Nerve**	**Ocular Globe**	**Retinal Atrophy**	**Liver / Kidney**
D42	Dec 1983	M/Juv		2	2	2	1	-	-	-	0/0
D65	1986	-/Ad		2	2	3	1 N2	-	-	-	-/-
D98[T]	Nov 1985	-/-		1	2	2	1	-	-	-	3/3
D123	Oct 1987	F/Ad		2	2	2	2 N3	-	-	-	0/0
D198	Jul 1989	F/Ad	Blind, dilated pupils, euthanised	4	2	2	3 N4	-	1	0	0/0
D228	Apr 1990	F/Ad	Depression, euthanised	1	1	-	1 N2	-	-	-	0/-
D229	May 1990	F/Ad	Depression, poor response to stimuli	3	4	2	1	-	-	-	0/0
D237	May 1990	F/Ad	Blind, euthanised	3	3	3	2 N1	1	0	3	-/-
D273[T]	1985	-/-	Emaciation	2	2	1	0	-	-	-	2/2
D275	May 1991	M/Ad	Blind, euthanised	3	3 N1	2	-	3	2	0	-/-
D284	May 1991	-/-	Blind, euthanised	2	2	2	2	2	2	4	-/-
D285	May 1991	-/-	Blind, euthanised	2	1	1	1 N3	-	-	-	-/-
D292	Nov 1991	F/Ad	Blind, euthanised	2	1	0	0	2	2	4	0/-
D293	Nov 1991	F/Ad	Blind	4	2	3	2	0	0	2	-/-
D294	Nov 1991	F/Ad	Blind, leg injury	1	2	2	1 N4	3	2	3	-/-
D305	Mar 1992	F/Ad	Blind several months, euthanised	4	4	4	-	-	-	-	0/-
D308	Mar 1992	F/Ad	Unilateral blindness, strabismus, euthanised	2	2	1	1 N3	-	-	-	1/-
D310	Feb 1992	F/Ad	Thin, rapid respiration	3	2	3	3 N3	1	1	4	0/0
D326	Jul 1992	F/Ad	Blind, ataxic, euthanised	3	2	1	2	-	-	-	1/-
D340	Aug 1992	F/Ad	Blind, ataxic	2	1	1	1	1	0	2	-/-
D365	Aug 1993	M/Ad	Blind	1	0	1	1	0	0	3	0/0
D372	Oct 1993	F/Ad	Blind, depressed, poor response to stimuli	5	4	4	2 N3	0	0	2	-/-

D375	1993	F/Juv	Blind, paralysis	2	1	1	1	-	0	4	-/-
D500	1997	F/Ad	Ataxic	1	0	0	1 N2	1	0	0	1/1
D503	1997	F/Ad	Blind	3	3	2	4	0	0	4	0/0
D541	1998	F/Ad	Blind, anisocoria, euthanised	3	3	3	3	4	4	4	0/0
D546	1998	F/Ad	Blind, thin, euthanised	4	3	3	3	1	2	1	0/0
1411.1	Mar 1993	F/Ad	Blind, progressive ataxia, weight loss, euthanised after 4 months	2	2 N2	2	1	1	0	4	0/0
1834.1	Aug 2000	F/Ad	Blind, euthanised after 6 weeks	3	2 N1	2	1	1	0	4	1g/1
2545.1	Nov 2001	M/Ad	Blind, nystagmus, thin, euthanised	1	1	1 N1	1	-	-	-	0/0
2723.1	Mar 2002	F/Ad	Blind, euthanised	0	0	0	0	0	2	1	1/-
2905.1	June 2002	F/Ad	Blind, euthanised	2	2	2	1	1	0	1	0/0
3046.1	Aug 2002	M/Ad	Blind, circling, ataxic, euthanised	2	1	2	1	2	3	4	0/0
3501.1	Apr 2003	M/Ad	Blind, knuckling, thin, euthanised	2	1	2	1	1	1	3	0/0
3619.1	Jul 2003	F/Ad	Blind, head low, slow, euthanised	2	1	2	2	3	0	4	1/1
4182.1	Jun 2004	F/Ad	Blind, ataxic, thin, euthanised	3	2	2	2	-	0	0	0/0
4231.1	Jul 2004	M/Ad	Circling, hit by car, euthanised	2	1	2	1	-	2	2	0/0
4601.1	Mar 2005	F/Ad	Blind, ataxic, thin, diarrhoea, euthanised	3	3	3	3 N2	-	-	-	0/0
5263.1	Jun 2006	F/Ad	Blind, circling, euthanised	2	3	2	2	-	0	3	0/0
6950.1	May 2009	M/Ad	Blind, depressed, penetrating wound, euthanised	2	2	2	1 N1	-	2	1	2**/1
6872.1	Mar 2009	F/Ad	Blind, thin, docile, euthanised	2	3	3	3	2	2	3	0/0
7635.1	Jun 2010	F/Ad	Blind, thin, euthanised	3	0	0	0	2	0	0	0/0
8554.1	Apr 2012	F/Ad	Blind, euthanasia	3	3 N2	3 N1	3 N1	2	2	0	0/1
8708.1	Jun 2012	M/Ad	Gasping, agonal, euthanised	2	3	2	2	-	-	-	0/0
8694.1	Jun 2012	F/Ad	Blind, ataxic, dead pouch young, euthanised	3	2	2	2	0	1	0	0/0
10282.1[T]	Dec 2014	F/Ad	Ataxic, euthanised	2	1	1	1	-	-	-	2/2
10282.2[T]	Dec 2014	F/Ad	Ataxic, euthanised	1	2	2	2	-	-	-	3/3
10282.2b[T]	Dec 2014	-/Juv	Euthanised with dam	1	2	2	2	-	-	-	2/3
10602.1	Jun 2015	F/Ad	Unresponsive, euthanised	1	1	1	1	1	0	0	1/0

All cases originate from NSW except Tasmanian cases that are denoted with a[T]
F- Female, M- Male, Ad – Adult, Juv - Juvenile

Non-suppurative inflammation graded on a scale of 0-4 in ascending severity. Necrosis signified by N and graded on a scale of 0-4 in ascending severity. – No sample.
[g] – granulomatous inflammation. ** associated with a penetrating foreign body.
